# A Clinic Trial Evaluating the Effects of Aloe Vera Fermentation Gel on Recurrent Aphthous Stomatitis

**DOI:** 10.1155/2020/8867548

**Published:** 2020-12-05

**Authors:** Yan Shi, Kehong Wei, Jiachen Lu, Jing Wei, Xiaojing Hu, Tingtao Chen

**Affiliations:** ^1^The Key Laboratory of Oral Biomedicine, Department of Conservative Dentistry and Endodontics, The Affiliated Stomatological Hospital of Nanchang University, Nanchang, China; ^2^School of Stomatology, Nanchang University, Nanchang, China; ^3^National Engineering Research Centre for Bioengineering Drugs and the Technologies, Institute of Translational Medicine, Nanchang University, Nanchang, China

## Abstract

Recurrent aphthous stomatitis (RAS) is the most common disorder in the oral mucosa that affects the daily quality of life of patients, and there is currently no specific treatment. In the present study, we developed aloe vera fermentation gel under the action of probiotics on aloe vera. In total, 35 patients with the history of aphthous stomatitis were enrolled to explore the potential benefits of aloe vera fermentation gel to treat RAS, and the healing-promotion effects were recorded and compared; microbial compositions in different groups were tested by high-throughput sequencing. Our results indicated that the duration of healing time of the aloe group showed potentially better effects because of the higher proportion of 4–6 day healing time (35% vs. 20%) and lower proportion of 7–10 day healing time (65% vs. 80%) compared with that of the chitosan group. Also, the use of aloe vera fermentation gel could return oral bacteria to normal levels and reduce the abundance of harmful oral bacteria including *Actinomyces*, *Granulicatella*, and *Peptostreptococcus*. These results suggest that aloe vera fermentation gel has the ability to treat patients with RAS and has positive prospects in clinical applications.

## 1. Introduction

Recurrent aphthous stomatitis (RAS) is a common disorder characterized by single or multiple ulcers with clear boundaries usually at the lingual margin, cheek, and lip; RAS affects 10–20% of the population and can heal automatically in about 10 days. Despite the high prevalence, the aetiological mechanism is not completely clear at present, but several risk factors, e.g., genetic susceptibilities, immune disorders, infections, vitamin and trace element deficiencies, systemic diseases, hormonal imbalances, mechanical damage, and stress, have been shown to be associated with the occurrence and progression of this disease [1, 2]. RAS has seriously affected the daily quality of life of patients (speaking, eating, and swallowing), but there is still no specific curative management for RAS, although topical medications, consisting of preservatives and anti-inflammatory/analgesics, aim to reduce pain degree and inflammation, while promoting disease healing [3]. Therefore, it is necessary to develop an effective agent for the treatment of RAS.

The oral cavity harbours many distinct microbial communities dominated by Firmicutes, Actinobacteria, Proteobacteria, Fusobacteria, and Bacteroidetes; disturbances of the oral microbiota contribute to the prognosis of a series of oral diseases, including mucositis and periodontitis and may be related to the aetiopathogenesis of RAS [4–6]. Given this, the intervention of saliva microbiota composition could play a potential role in the treatment of RAS [7–9]. In recent years, the concept of probiotics has gradually entered people's vision, has been used in the treatment of various oral diseases such as periodontitis and mucositis, and can be used as an adjunctive in treating RAS [10–13]. Aloe vera is a cactus-like plant that has been broadly used as a cosmetic moisturiser, toothpaste, food flavouring, and preservative in the pharmaceutical and food fields; it can also be used in medicine for its effects such as wound healing, anti-inflammation, antioxidant, antibacteria, antifungal, antiviral, and antitumour properties [1, 14]. Moreover, previous studies indicated that aloe vera showed the ability to treat RAS evidenced by reduced pain and healing time [1].

Considering the therapeutic efficacies of aloe vera and probiotics, the probiotic fermentation products of aloe vera show promise in RAS treatment, as fermentation by probiotics can result in new compounds which have the potential for health-modulation and produce lots of metabolites including lactic acid, an antioxidant that can strengthen the beneficial effects of probiotics and aloe vera [15]. Moreover, our previous studies demonstrated that aloe vera fermentation showed strong antibacterial, antioxidant, and anti-inflammatory activities and possessed a strong burn injury healing effect in vivo [14, 16].

In the present study, the aloe vera fermentation gel was produced and used for RAS patients to evaluate its effects on shortening the healing time and restoring microbial diversity in the oral cavity.

## 2. Materials and Methods

### 2.1. Aloe Vera Fermentation Gel Preparation

*Lactobacillus plantarum* MH-301 (provided by Harbin Meihua Biotechnology Co, Ltd, Harbin, Heilongjiang, PR China) was cultured in 5 ml Man–Rogosa–Sharpe (MRS) medium 10–16 h for static cultivation at a temperature of 37°C. Then, *L. plantarum* MH-301 was cultured in fresh MRS medium for amplification cultivation overnight at 37°C. The aloe vera leaves were cut off and mashed after being washed thoroughly. Sterile water (1 : 1) and edible glucose (5% of aloe vera mass) were added to the mashed aloe vera. The overnight cultured probiotic was centrifuged at 8000 g for 5 min and washed with sterilised phosphate-buffered saline (PBS) 2–3 times; the probiotics were resuspended by adding them to sterilised PBS at a level of 5% of aloe vera. The probiotics were inoculated into the inactivated aloe vera solution and incubated for 36–72 h at 37°C for fermentation until the pH reached 3–4. The aloe vera fermentation gel was prepared by adding gelatine powders to the aloe vera solution and conserved at 4°C.

### 2.2. Ethical Statement

This trial was conducted at the Affiliated Stomatological Hospital of Nanchang University, China, in 2019. The trial was approved by the Ethical Committee of Affiliated Stomatological Hospital of Nanchang University (No. 2019–008) and had been registered at the Clinical Trail Registry (identifier: ChiCTR1900023903). All participants signed a written informed consent form, and all methods were performed in accordance with the approved guidelines.

### 2.3. Participants and Selection Criteria

Forty-six patients were enrolled, and 35 patients (12 male and 23 female) aged 18–60 years were selected from the Affiliated Stomatological Hospital of Nanchang University patients, and an additional 10 healthy people were selected as the negative control. All patients were enrolled 2–5 days after the occurrence of first oral ulcer. Inclusion criteria consisted of the following: (a) clinical examination and confirmed history of recurrent oral ulcer, (b) recurrence of the oral ulceration, and (c) patients in good condition with no serious systemic disease. Eligible persons were excluded if they were administered antibiotics or glucocorticoids or accepted periodontal or dental treatments that interfered with the results of our experimental drugs.

### 2.4. Trial Design

The baseline information of patients, including individual information (age and gender) and characteristics of ulcers (duration of healing time), were reported for all patients. Oral inspections were conducted by one inspector of the Affiliated Stomatological Hospital of Nanchang University. All patients were divided into two groups. For the first group, patients were applied with aloe vera fermentation gel, reported as the AA group. The remaining patients were using chitosan gel (AC group). All patients were informed to apply a layer of gel on the surface of the ulcer every day after each meal (three times each day) until the ulcer disappeared. The duration of recovery for all patients in the two groups was recorded. The saliva of patients was collected before the use of gels as a positive control (PC group) and collected again after the use of drugs once the ulcers disappeared. Additionally, 10 healthy individuals were enrolled into this experiment as the negative control group (NC group) and their saliva was collected and conserved for further high-throughput sequencing.

### 2.5. DNA Extraction and High-Throughput Sequencing

Saliva samples were taken and stored at −80°C. The combination of genomic DNA kits and a bead method was used, and the concentration and quality of DNA were determined by ultraviolet photometer. The V4 regions of the 16S rDNA genes of each sample were amplified by PCR amplification using designed primers of 515F/806R (515F, 5′-GTGCCAGCMGCCGCGGTAA-3′; 806R, 5′-GGAC TACVSGGGTATCTAAT -3′) according to the conserved regions in the sequence. Amplified DNA products were sequenced by an Illumina MiSeq platform, and the raw data were conserved in the form of FASTQ (Gene Bank accession number PRJNA656084).

### 2.6. Data Analysis

Paired-end reads from the original DNA fragments were processed by FLASH software (v1.2.7, http://ccb.jhu.edu/software/FLASH/) and QIIME software (v1.8.0, http://qiime.org/). Sequences with ≥97% similarity were regarded as the same operational taxonomic units (OTUs). The compositions and relative abundances of each sample at the phylum level and the genus level were analysed using the QIIME software. The QIIME software (v1.8.0) was also used to analyse the *a*-diversity (within samples, indexes of observed OTUs) and *ß*-diversity (between samples, PCoA). According to the obtained OTU abundance matrix, the total number of OTUs in each sample (group) was calculated and visualised through the Venn diagram using *R* software.

All data were reported as mean ± SD, and results were analysed using SPSS20.0 (Chicago, IL) and GraphPad Prism (v6.0) via one-way ANOVA, chi-squared test, and unpaired *t* test. All tests were two-tailed, and the *p* value of 0.05 was considered to be statistically significant.

## 3. Results

### 3.1. Participants

**Figure 1:**
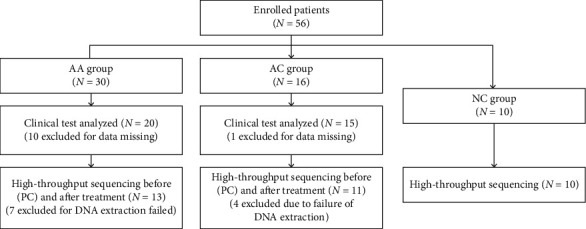
Flow diagram of the trial; forty-six patients with RAS were enrolled into our trial, and then 11 patients (10 in AA and 1 in AC) were excluded due to data missing from the clinical test analysis. Moreover, 11 patients (7 in AA and 4 in AC) were excluded from high-throughput sequencing analysis as the DNA extraction failed. Additionally, 10 healthy individuals were selected for high-throughput sequencing as a negative control group.

A total of 46 volunteers (16 male and 30 female) were enrolled in the study. During the clinical test analysis, 10 in the AA group and 1 in the AC group were excluded due to missing data. Subsequently, 7 in the AA group and 4 in the AC group were excluded as the DNA extraction failed. Additionally, 10 healthy individuals were enrolled for high-throughput sequencing. The flow diagram is shown in Figure 1.

### 3.2. Aloe Vera Fermentation Gel Accelerated the Healing of RAS

The AA and AC groups were fixed by age and gender (*p*=0.186, *p*=0.411, respectively). The mean healing time for aloe vera fermentation gel and chitosan gel was 7.40 ± 1.85 days and 7.93 ± 1.84 days, respectively. Additionally, all patients were healed 4–10 days after the occurrence of ulcers; the healing time was divided into 4–6 days and 7–10 days to analyse the healing effects in the AA and AC groups based on clinical experience. The proportion of patients with a 4–6 day healing time in the AA group was higher than in the AC group (35% vs. 20%, respectively; *p*=0.728), whereas the proportion of patients with a 7–10 day healing time in the AA group was lower than in the AC group (65% vs. 80%m respectively; *p*=0.931) (Table 1). Thus, aloe vera fermentation gel had a potentially better wound healing effect than chitosan gel.

### 3.3. The *α*- and *ß*-Diversities of the Oral Microbial Community

**Figure 2:**
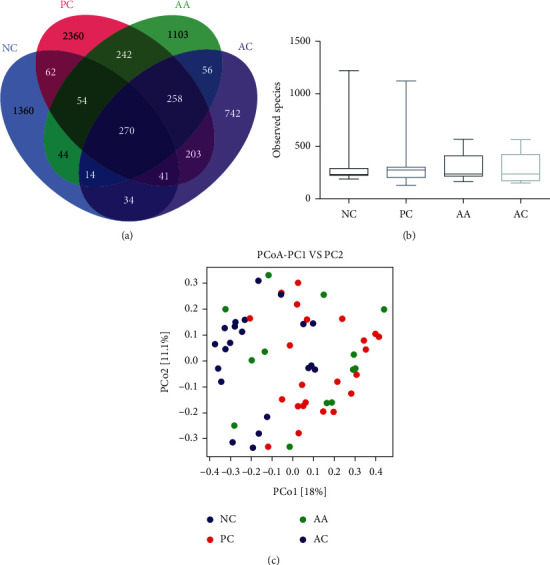
Evaluation of aloe vera fermentation gel on *α*-diversity (within samples), *β*-diversity (between samples), and Venn diagram representation. (a) Common OTUs' Venn diagram. (b) Observed species. (c) Principal coordinates analysis (PCoA).

From the 58 communities in the NC, PC AA, and AC groups, 6,345,619 filtered clean tags (average of 109,407.22 filtered clean tags per sample) and 18,739 OTUs (average of 323.09 OTUs per sample) were obtained from all samples (data not shown). The common OTU Venn diagram suggested that there are 270 common core OTUs found in all groups; the numbers of unique OTUs in the NC, PC, AA, and AC groups was 1,360, 2,360, 1,103, and 742, respectively (Figure 2(a)). As shown in Figure 2(b), observed species were analysed to estimate the *α*-diversity of the bacterial communities; no significance was observed in the NC (336.86 ± 294.46), PC (301.89 ± 195.29), AA (304.68 ± 130.14), and AC (283 ± 132.50) groups. The principal coordinates analysis (PCoA) was used to evaluate the OTU relationship between different groups (Figure 2(c)), and the microbial diversities in the PC group were significantly different from those in the NC group; treatment with the aloe vera fermentation gel could alter the microbial diversities toward to the NC group.

### 3.4. Comparison of the Oral Microbiota Composition

**Figure 3:**
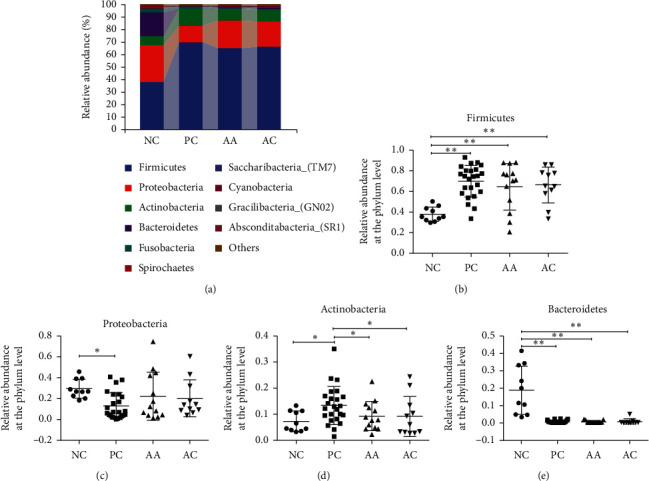
Evaluation of the aloe vera fermentation gel on the bacterial compositions at the phylum level. (a) The relative abundances of the top 10 bacteria at the phylum level. The relative abundances of (b) Firmicutes, (c) Proteobacteria, (d) Actinobacteria, and (e) Bacteroidetes. All data are shown as mean ± SD. Significant differences are denoted by ^*∗*^*p* < 0.05 and ^*∗∗*^*p* < 0.01.

**Figure 4:**
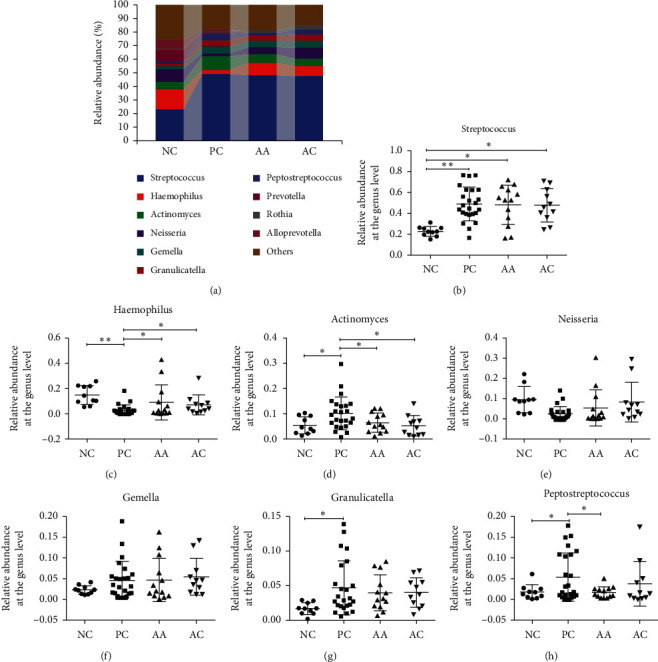
Evaluation of the aloe vera fermentation gel on the bacterial compositions at the genus level. (a) Taxonomic profiles at the phyla level in 58 saliva samples of the top 10 genera at the genus level. The relative abundances of (b) *Streptococcus*, (c) *Haemophilus*, (d) *Actimomyces*, (e) *Neisseria*, (f) *Gemella*, (g) *Granulicatella*, and (h) *Peptostreptococcus*. All data are shown as mean ± SD. Significant differences are denoted by ^*∗*^*p* < 0.05 and ^*∗∗*^*p* < 0.01.

As shown in Figure 3, the relative abundance of bacteria was compared between the NC, PC, AA, and AC groups. The results suggested that the bacteria from the genera Firmicutes, Proteobacteria, Actinobacteria, and Bacteroidetes were dominant and comprised >93% of oral bacteria in all groups at the phylum level. Additionally, we analysed the relative abundance of Firmicutes, Proteobacteria, Actinobacteria, and Bacteroidetes, respectively. The results showed that the abundance of Firmicutes (37.98% vs. 69.87%) and Actinobacteria (7.33% vs. 13.44%) were increased in the PC group compared with that in the NC group, whereas the abundance of Proteobacteria (29.34% vs. 12.84%) and Bacteroidetes (18.81% vs. 0.64%) was decreased in the PC group. After treatment, the relative abundance of Proteobacteria (22.14%) and Actinobacteria (9.32%) was changed toward to the normal level in the AA group. At the genus level, the ten most abundant genera were Streptococcus, Haemophilus, Actinomyces, Neisseria, Gemella, Granulicatella, Peptostreptococcus, Prevotella, Rothia, and Alloprevotella, accounting for >74% of all bacteria (Figure 4). In the PC group, the relative abundance of Streptococcus (22.85% vs. 48.97%), Actinomyces (5.40% vs. 10.10%), Gemella (2.30% vs.4.63%), Granulicatella (1.70% vs. 4.70%), and Peptostreptococcus (1.78% vs. 5.59%) was increased compared with that of the normal NC group, while the abundance of Haemophilus (14.75% vs. 2.83%) and Neisseria (9.48% vs. 2.57%) was decreased in the PC group compared with that of the NC group. The use of aloe vera fermentation gel (Haemophilus (8.96%), Actinomyces (6.43%), Neisseria (5.39%), Granulicatella (3.98%), and Peptostreptococcus (1.63%)) returned to normal levels in the AA group.

## 4. Discussion

RAS causes pain and difficulties with eating, speaking, and swallowing, thereby affecting the patients' quality of life [17]. In consideration of the beneficial effects and safety of herb medicines, it is meaningful to develop a new herb medicine in the treatment of RAS.

In the present study, we developed the aloe vera fermentation gel, and its effects on RAS were evaluated and compared with the approved chitosan gel on the market in 35 patients from the Affiliated Stomatological Hospital of Nanchang University. The results suggested that the oral ulceration in these patients could disappear within 10 days, with 35% of patients recovered within 4–6 days and 65% of patients recovered by 7–10 days. However, only 20% of patients using chitosan gel were recovered within 4–6 days, and 80% of patients were recovered by 7–10 days (Table 1), indicating that the aloe vera fermentation gel had potentially better wound healing benefits than chitosan gel.

For RAS, wound healing and anti-inflammation are important for patients. Aloe vera is a cactus-like plant that has been widely used in medicine to treat burn injuries, cutaneous wounds, and oral ulceration, which makes it a good candidate for the treatment of RAS [18, 19]. The reasons for the shortened healing time in the AA group may be due to the effectiveness of aloe vera for promoting wound healing and anti-inflammation and the strong anti-inflammatory, immunomodulatory, antioxidative, and antibacterial effects endowed by probiotics during aloe vera fermentation [20, 21]. RAS is a chronic inflammatory disease, and the benefits of aloe vera have been shown in the treatment of this disease [22]. Inflammation is a dynamic process with proinflammatory cytokines, and aloe vera shows anti-inflammatory benefits by inhibiting inflammatory processes and proinflammatory cytokines [23]. For example, Aloe vera can inhibit the leukocyte infiltration, eicosanoid formation, and generation of inflammatory mediators including histamine and bradykinin [1]. Aloe vera contains a series of components such as acemannan which have wound healing potential by enhancing the repair process and epithelial cell proliferation via the induction of factors contributing to wound repair including fibroblasts and collagen [1, 24]. Furthermore, the immunomodulatory effects of aloe vera also suggest its potential benefit for RAS treatment [1, 19]. The antioxidant components in aloe vera also enhance the anti-inflammatory effects by inhibiting the production of reactive oxygen metabolites, therefore preventing oxidative stress [25]. Probiotics are live microorganisms that can confer a healthy benefit on the host and can be used as an adjunctive in various diseases due to wound healing effects and antimicrobial effects against various pathogens [13, 26]. The topical use of probiotics can be used as an antagonist against wound pathogens and enhance wound healing effects, by decreasing the pathogen load, and can be used in the treatment of burn infections and chronic ulcers [26]. Immune system dysfunction favours the occurrence of inflammatory reactions and the appearance of RAS [27]. Evidence indicates that probiotics can modulate the immune response along with anti-inflammation to influence the progression of RAS [27]. The anti-pathogenic properties, together with tissue repair, and immunomodulatory, and anti-inflammatory properties of probiotics, make them an attractive option in RAS [28]. In particular, the combination of aloe vera and probiotics during aloe vera fermentation confers a better effect.

The human oral cavity belongs to the second-most abundant source of microbiota after the gastrointestinal tract, and previous studies have shown that the oral microbiota in healthy individuals are different from the microbiota observed in patients with various oral diseases and oral dysbiosis [6, 29–31]. Our results suggest that the aloe vera fermentation gel can not only shorten the healing time but also alter the speed at which bacterial compositions return to normal levels (Figures 3 and 4). The relative abundances at the phylum level were analysed; the first six phyla composed of Firmicutes, Proteobacteria, Actinobacteria, Bacteroidetes, Fusobacteria, and Spirochaetes were consistent with those in the previous studies of oral microbiota [6]. We found that Firmicutes was significantly increased, whereas Proteobacteria and Bacteroidetes were significantly reduced in RAS patients. The relative abundances of Actinobacteria were increased in the PC group and decreased after treatment with aloe vera fermentation gel, which indicates that this drug has potential benefit with regard to returning relative abundances to normal levels. The relative abundances at the genus level were also compared between groups. The decreased *Haemophilus* and *Neisseria* and increased *Actinomyces*, *Granulicatella*, and *Peptostreptococcus* in the PC group and normalised abundances in the AA group demonstrated that aloe vera fermentation gel had benefits in the maintenance of microbial compositions [32]. Additionally, the decreased *Haemophilus* and *Nesseria* correlated the results of a previous study showing that decreased Proteobacteria, containing *Haemophilus* and *Nesseria*, is related to oral diseases such as gingivitis and cancer [33–36]. *Actinomyces* has long been recognised as a causative agent of actinomycosis as it increases the pathogenicity by attacking broken or necrotic tissues and is related to the incidence of RAS, with high abundance indicating poor prognosis for RAS [37, 38]. Also, the previous studies have shown that *Granulicatella* is raised in patients with oral inflammatory diseases [39, 40]. From the community alternations, we concluded that ulceration could change the bacterial compositions and lasts for the entire ulcerative process. Nonetheless, the intervention of aloe vera fermentation gel could normalise some of the bacteria at the phylum and genus levels and decrease the abundance of harmful oral bacteria which indicate good prognosis and suggest that our new drug has the potential to be used in the clinical setting.

In conclusion, this clinical trial showed that Aloe vera fermentation gel effectively facilitated the healing process and normalised microbiota disorders of RAS. It can reduce the abundance of harmful oral bacteria including *Actinomyces*, *Granulicatella* and *Peptostreptococcus*, which implies a better prognosis. Therefore, it could improve the quality of life for patients with RAS. This offers a direction for future research, and provides a potential drug for clinical use. However, the effects of Aloe vera fermentation gel were evaluated in just two aspects, so further underlying mechanism tests are needed to accelerate the clinical application of this drug.

## Figures and Tables

**Table 1:** Patients' baseline information and characteristics.

Variable	AA group	AC group	*p* value
Percentage of total enrollment, No (%)	20 (57.14)	15 (42.86)	—
Male; female, n: *n* (%: %)	8 : 12 (40.00 : 60.00)	4 : 11 (26.67 : 73.33)	0.411
Age, y	30.5 y26.6	26.0756.44	0.186
Duration of healing time, no. (%)			
4–6 days	7 (35.00)	3 (20.00)	0.728
7–10 days	13 (65.00)	12 (80.00)	0.931

AA, patients treated with aloe vera fermentation gel. AC, patients treated with chitosan gel.

## Data Availability

The deidentified raw data used to support the findings of this study are available from the corresponding author upon request.
